# Supplementary material for: Evaluation of Antiviral Activity of Ivermectin against Infectious Bovine Rhinotracheitis Virus in Rabbit Model
Source: Animals (Basel). 2023 Oct 10;13(20):3164. doi: 10.3390/ani13203164 (PMC10603647; doi:10.3390/ani13203164)
Supplement: Supplementary file 1 [file animals-13-03164-s001.zip › animals-2601561-supplementary.pdf]

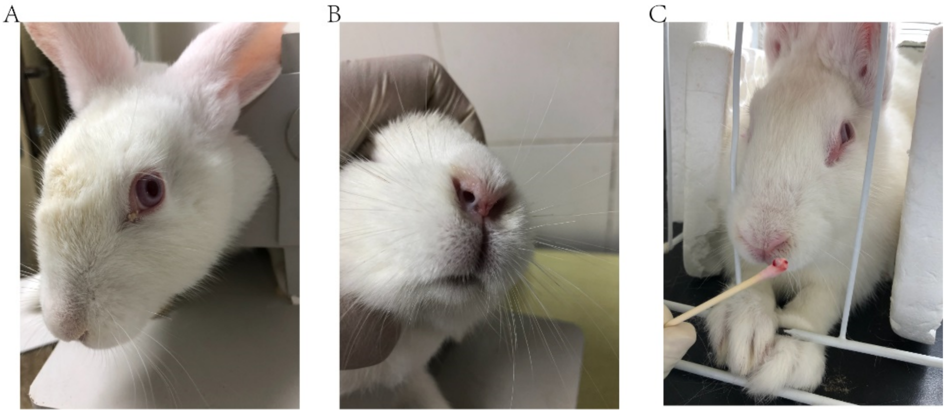

Figure S1. Clinical symptoms in rabbits after infection. A: Purulent discharge from the eyes; B: Purulent discharge from the nasal; C: Nasal mucosal bleeding accompanied by purulent discharge.

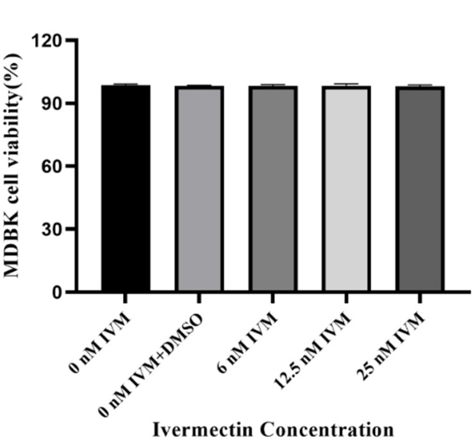

Figure S2. IVM does not affect cell viability. MDBK cells were incubated with increasing concentrations of Ivermectin for 48 h. Cell viability was then measured using an MTT assay. Means ±SD of n=3 independent experiments, unpaired Student's t-test.

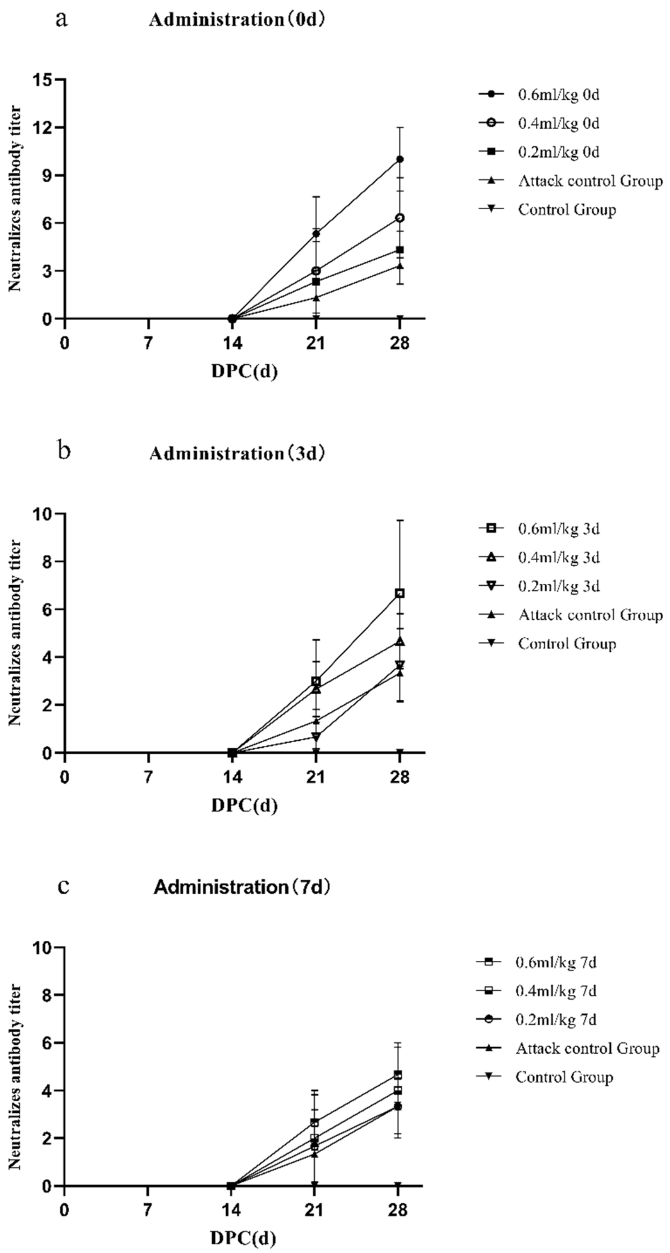

Figure S3. Neutralization antibody level at different time points of administration. a: Neutralization antibody levels in different dose groups when administered on 0 d; b: Neutralization antibody levels in different dose groups when administered on 3 d: c: Neutralization antibody levels in different dose groups when administered on 7 d.
